# Real world outcomes with alpelisib in metastatic hormone receptor-positive breast cancer patients: A single institution experience

**DOI:** 10.3389/fonc.2022.1012391

**Published:** 2022-10-20

**Authors:** Sabah Alaklabi, Arya Mariam Roy, Kristopher Attwood, Anthony George, Tracey O’Connor, Amy Early, Ellis G. Levine, Shipra Gandhi

**Affiliations:** ^1^ Department of Medicine, Roswell Park Comprehensive Cancer Center, Buffalo, NY, United States; ^2^ Department of Biostatistics & Bioinformatics, Roswell Park Comprehensive Cancer Center, Buffalo, NY, United States; ^3^ Department of Immunology, Roswell Park Comprehensive Cancer Center, Buffalo, NY, United States

**Keywords:** alpelisib, piqray, PIK3CA, breast cancer, real-world, effectiveness, adverse events

## Abstract

**Background:**

It is critically important to study the real-world data of FDA-approved medications to understand the response rates and toxicities observed in the real-world population not represented in the clinical trials.

**Methods:**

We reviewed charts of patients diagnosed with metastatic, hormone receptor-positive, human epidermal growth factor receptor 2 negative, PIK3CA-mutated breast cancer treated with alpelisib from May 2019 to January 2022. Clinical characteristics and treatment outcomes were collected. The association of clinical characteristics with responses and adverse events (AEs) was evaluated using the logistic regression model.

**Results:**

27 patients were included. Median age at alpelisib initiation 67 years (range: 44, 77 years). Majority of patients had excellent performance status at time of alpelisib initiation. Most patients had chronic comorbidities, notably; 2 patients had controlled type 2 diabetes mellitus at time of alpelisib initiation. Majority had a median of three lines of therapy (range: 1, 7) before alpelisib. Clinical responses were determined using RECIST v1.1. 3/27 (11.11%) patients discontinued therapy before response assessment due to grade 3 AEs. Overall response rate was 12.5% (3/24), with all partial responses (PR). The median duration of response was 5.77 months (range: 5.54, 8.98). 14/27 (51.9%) of patients required dose interruption/reduction. Overall, 23/27 (85.19%) patients discontinued alpelisib of which 11 (47.83%) discontinued alpelisib due to AEs. Median duration of treatment was 2 months in patients who had grade 3 AEs (range: <1.00, 8.30) and 6.28 (1.15, 10.43) in those who did not. Any grade AEs were reported in 24/27 (88.9%) patients, namely, hyperglycemia 16/27 (59.3%), nausea 11/27 (40.7%), diarrhea 10/27 (37.0%), fatigue 7/27 (25.9%) and rash 6/27 (22.2%). Grade 3 AEs were reported in 13/27 patients (50%), namely, hyperglycemia in 7/27 (53.8%) patients followed by skin rash 4/27 (30.8%), GI side effects 3/27 (23.1%). Those with progressive disease as best response to alpelisib, had more non-metabolic comorbidities, higher number of liver metastases, PIK3CA E545K mutations, and shorter duration on therapy compared to those with PR and stable disease.

**Conclusion:**

Patients should be counseled about the toxicity and modest benefit observed with alpelisib in real-world clinical practice when used in later lines of therapy.

## Introduction

Breast cancer is the most common cancer diagnosed among women and the second leading cause of cancer deaths in the United States. Breast cancer in women is the fifth leading cause of death worldwide. Women with metastatic breast cancer have a 5-year overall survival rate of 29% (1). Hormone-receptor (HR) positive human epidermal growth factor receptor (HER 2) negative is the most prevalent subtype, with 88.1 new cases per 100,000 women during 2014–2018 per the SEER database (1). Over the years, although several medications have been approved, there has been a lack of targeted drugs in this space. Alpelisib targets PIK3CA gene mutation which is identified in up to 40% of HR-positive HER2-negative primary and metastatic breast cancer (2). It has received FDA approval in May 2019 for patients with metastatic HR-positive breast cancer with PIK3CA mutation who have received prior endocrine therapy. The approval was based on SOLAR 1, a phase 3, randomized, double-blind, placebo-controlled trial that examined the efficacy and safety of alpelisib plus fulvestrant versus placebo plus fulvestrant in postmenopausal women, and men, with HR-positive, HER2-negative, advanced, or metastatic breast cancer whose disease had progressed on or after receiving an aromatase inhibitor. The primary endpoint was progression-free survival (PFS) which was achieved with a median of 11.0 months in PIK3CA mutations carriers compared to 5.7 months in the placebo arm. However, overall survival (OS) data was not statistically significant (3, 4). Patients who had previously received a cyclin-dependent kinase 4/6 (CDK4/6) inhibitor were a minority in SOLAR-1. Hence it was critical to determine whether patients who had previously received a CDK4/6 inhibitor would benefit from alpelisib without concerning safety signals. To answer that question, another clinical trial, BYLieve was conducted which was a phase 2, open-label, non-comparative study that enrolled patients with HR-positive, HER2-negative, advanced breast cancer with tumor PIK3CA mutation, following progression on or after previous therapy, including CDK4/6 inhibitors with no more than two previous anticancer treatments and no more than one previous chemotherapy regimen. The study concluded that this population still derived benefit with a comparable safety profile to what was reported in SOLAR-1 (5, 6).

The development of alpelisib expanded the nonchemotherapy alternatives for patients with HR-positive HER2-negative metastatic breast cancer and exemplified the trend in cancer care toward personalized medicine. Since its approval, however, there have been strong arguments to withdraw the drug from the market given concerns about the drug’s tolerance and effectiveness in real-world clinical practice (7). As is well known, clinical trials select a healthier pool of patients compared to patients we actually see in the clinic to whom we often extrapolate results of clinical trials. The goal of this study was to investigate the real-world outcomes among patients treated with alpelisib post-FDA approval in an NCI-designated cancer center. The aim is to provide healthcare practitioners with more information so they can make better decisions when prescribing alpelisib in certain situations.

## Materials and methods

The Roswell Park Institutional Review Board (IRB) reviewed and approved the research protocol (IRB 144321). This single-institution study included patients treated at Roswell Park Comprehensive Cancer Center in Buffalo, New York. From June 2019 to January 2022, electronic records of patients with HR-positive HER2 negative, PIK3CA-mutated metastatic breast cancer who were treated with alpelisib in combination with fulvestrant or an aromatase inhibitor were retrospectively analyzed. Alpelisib-treated metastatic breast cancer patients’ medical records were accessed using an institutional search engine. Two investigators (SA and AR) subsequently went through the medical records and performed an in-depth hand-review of each one to ascertain the demographic and clinical characteristics of the patients, their comorbidities, and the outcome data while on alpelisib treatment. Baseline demographic characteristics included age, race, body mass index (BMI), clinical characteristics included comorbidities, site, and the number of visceral metastases, prior lines of treatment in the metastatic setting, time from initiation of alpelisib until progression, and last follow-up. In addition, investigators evaluated tumor assessment according to the Response Evaluation Criteria in Solid Tumors version 1.1 (RECIST v1.1) at the intervals decided by treating physicians which are mostly based on clinical judgment (8).

### Sample size, statistical methods, and analyses

The number of patients with metastatic breast cancer who had been administered alpelisib outside of a clinical trial at the time of data collection defined the sample size; the current study included all eligible patients. The data was locked in January 2022. All statistics were performed using SAS version 9.4 (SAS Institute Inc., Cary, NC).

Study characteristics were stratified by best response, objective response rate (ORR) defined as the percentage of patients with complete response (CR) or partial response (PR) based on the study investigator’s (SA and AR) personal review of scans according to RECIST v1.1. The frequencies and relative frequencies for best response and adverse events were reported. The mean, median, standard deviation, and range were provided for continuous variables and analyzed using the Mann-Whitney U test, and the Kruskal-Wallis test for the best response outcome measure. The frequencies and relative frequencies were provided for categorical variables and analyzed using Fisher’s exact test.

Patients who were still alive until January 2022 (data cut off) were censored for survival. Standard Kaplan Meier (KM) methods were used to create survival curves illustrating the OS and PFS. OS was calculated using the log-rank test. OS was defined as survival from the start of alpelisib until the date of last follow-up or death. PFS is defined as the time between the date of the first dose to the date of first documented progression or death.

The relative dose intensity (RDI) was also explored further for its potential association with study survival. RDI is defined as the actual dose delivered divided by the standard dose (9–11). RDI is calculated as RDI = (DDI (delivered dose intensity)/SDI (standard dose intensity)) ×100%. RDI was dichotomized at the median and analyzed using KM curves for OS and PFS and log-rank p-values were reported. Data were analyzed using dichotomizations: RDI_median or < median.

## Results

### Demographic, laboratory, and treatment characteristics of the study population

Between May 2019 and January 2022, a total of 28 HR-positive HER2-negative metastatic breast cancer women with PIK3CA mutation received alpelisib. All were included except one patient as it was unclear from the chart review if she had ever started the drug. All of the patients in this cohort received alpelisib in combination with fulvestrant per SOLAR1.

The median age at the initiation of alpelisib was 67 years (range: 44, 77 years). Majority of patients were white 23/27 (85.2%), and had excellent performance status using the Eastern Cooperative Oncology Group (ECOG) performance status scale at the time of treatment initiation: ECOG = 0 in 15/27 (55.6%) and 1 in 12/27 (44.4%) patients. Most of the patients had chronic comorbidities, such as hypertension, cardiovascular, and thyroid disease (66.7%). Of note, 2 patients had controlled type 2 diabetes mellitus at time of initiation of alpelisib. Clinical characteristics are summarized in **Table 1**.

**Table 1:** Baseline clinical/demographic characteristics.

Variables		N = 27 (%)
Gender	Female	27 (100%)
Race	White	23 (85.2%)
	Black	1 (3.7%)
	Hispanic	1 (3.7%)
	Asian	1 (3.7%)
Age at initiation of alpelisib (years)	Median (range)	66.81 (43.70-77.32)
Mean BMI	Mean/Std	26.82/5.38
ECOG	0	15 (55.6%)
	1	12 (44.4%)
Comorbidities	No	9 (33.3%)
	Yes	18 (66.7%)
	Type 2 diabetes	2 (7.4%)
	Hypertension	9 (33.3%)
	Dyslipidemia	5 (18.5%)
	Cardiovascular disease	2 (7.4%)
	Thyroid disease	4 (14.8%)
	Chronic lung disease	2 (7.4%)
	*Nonmetabolic comorbidities	5 (18.5%)
Number of organ metastasis	1	2
	2	13
	3	9
	4	3
Site of metastasis	Bone metastasis	26 (96.3%)
	Brain metastasis	3 (11.1%)
	Liver metastasis	13 (48.1%)
	Lung metastasis	6 (22.2%)
	Visceral metastasis	19 (70.4%)
Prior best response	PR	6 (22.2%)
	SD	21 (77.8%)
Longest prior duration of response (months)	Median (range)	24.00 (4.00-114.00)
Histology	IDC	6 (22.2%)
	ILC	8 (29.6%)
	Poorly differentiated	12 (44.4%)
	Mixed	1 (3.7%)
**Molecular Factors and Mutations**		
Type of molecular test	Tissue molecular test	13 (48.1%)
	Peripheral blood	14 (51.9%)
Timing of molecular test	1st progression	6 (23.1%)
	2nd progression +	20 (76.9%)
PIK3CA mutation subtype	PIK3CA H1047R mutation	14 (51.9%)
	PIK3CA E545K mutation	9 (33.3%)
	PIK3CA E546K mutation	1 (3.7%)
	PIK3CA E542K mutation	3 (11.1%)
	PIK3CA E418K mutation	1 (3.7%)
	PIK3CA C420R mutation	1 (3.7%)
	PIK3CA D350N mutation	1 (3.7%)
Laboratory values at baseline	WBC (10^9^/L) median (range)	5.20(1.58-16.20)
	ALP (IU/L) median (range)	89.00 (21.00-725)

BMI, Body mass index; ECOG PS scale, Eastern Cooperative Oncology Group performance status scale; PR, partial response; SD, stable disease; IDC, invasive ductal carcinoma; ILC, invasive lobular carcinoma; WBC, white blood cells; ALP, alkaline phosphatase.

*Nonmetabolic comorbidities: one patient had history of venous thromboembolism; one patient had transient ischemic attack; one patient had leukemia; one patient had gastroesophageal disease.

Baseline white blood count (WBC) and alkaline phosphatase (ALP) values are summarized in **Table 1**. Most patients had had a median of three previous cancer therapy regimens (range: 1.00, 7.00) prior to alpelisib, which included prior cytotoxic therapy (eribulin, taxane, capecitabine, gemcitabine, platinum, anthracycline), targeted agents (CDK4/6 inhibitors, everolimus, olaparib), and hormonal agents (AI, selective estrogen receptor degrader or down-regulator (SERD) (**Table 2**).

**Table 2:** Prior lines of therapy in the metastatic setting.

Variables		N (%)
Median number of prior lines of therapy (range)		3 (1-7)
Prior targeted agents	CDK4/6 inhibitors + AI	20 (74.1%)
	CDK4/6 inhibitors + Fulvestrant	8 (29.6%)
	Everolimus + AI	11 (40.7%)
	Olaparib	2 (7.4%)
Prior clinical trial		2 (7.4%)
Prior cytotoxic therapy	Eribulin	2 (7.4%)
	Taxane	10 (37%)
	Xeloda	12 (44.4%)
	Gemcitabine	1 (3.7%)
	Platinum	1 (3.7%)
	Anthracycline	2 (7.4%)
	A/C	1 (3.7%)
Prior hormonal agents	Single-agent AI/SERD	9 (33.3%)

CDK4/6 inhibitors, cyclin-dependent kinase 4/6 inhibitors; AI, aromatase inhibitors; A/C, anthracycline and cyclophosphamide; SERD, selective estrogen receptor degrader.

Alpelisib was started in the 5^th^ line and beyond in a third of the patients 10/27 (37%) of patients, 3^rd^ line in 7/27 (25.9%), and 4^th^ line in 4/27 (14.8%) of patients. Only 6/27 (22.2%) patients received alpelisib as indicated by the FDA label in the second-line setting. The median dose at the time of initiation was 300 mg (range: 150, 300). 4/27 (14.8%) patients were still on alpelisib at the time of data cutoff.

### Histology and sites of metastases

Tumors from 12/27 (44.4%) patients had poorly differentiated histology. The median number of sites involved by metastasis was two (range: 1.00, 4.00). Notably, 26/27 (96.3%) patients had bone metastasis, 3/27 (11.1%) had brain metastasis, and 19/27 (70.4%) patients had visceral metastasis, of which 13/19 (68%) had liver metastasis, informing that this was a population with relatively aggressive disease (**Table 1**).

### Toxicity analysis

Any grade adverse events (AEs) attributed to alpelisib were reported in 24/27 (88.9%) patients. The most common being hyperglycemia 16/27 (59.3%), nausea 11/27 (40.7%), diarrhea 10/27 (37.0%), fatigue 7/27 (25.9%), and rash 6/27 (22.2%). Overall grade 3 AEs were reported in 13/27 (50%) patients with the most common being hyperglycemia in 7/27 (53.8%) patients followed by skin rash in 4/27 (30.8%), and gastrointestinal (GI) side effects in 3/27 (23.1%) (**Table 3**).

**Table 3:** Adverse events of alpelisib.

Adverse Events (AE)	N = 27 (%)	
No	3	
Yes	24	
	Hyperglycemia	16 (59.3%)
	Nausea	11 (40.7%)
	Decreased appetite	4 (14.8%)
	Rash	6 (22.2%)
	Vomiting	4 (14.8%)
	Diarrhea	10 (37%)
	Deceased weight	2 (7.4%)
	Stomatitis	5 (18.5%)
	Fatigue	7 (25.9%
	Cytopenia	2 (7.4%)
	Renal impairment	1(3.7%)
	Pneumonitis	1(3.7%)
	*Other AEs	1(3.7%)
**Grade 3 AEs**	**N = 13 (%)**	
	Hyperglycemia	7 (53.8%)
	Rash	4 (30.8%)
	Diarrhea	2 (15.4%)
	**N = 13 (%)**	
	Vomiting	1 (7.7%)
	Pneumonitis	1 (7.7%)
	Thrombocytopenia	1 (7.7%)

National Cancer Institute- Common Terminology Criteria for Adverse Events v5 (NCI-CTCAE), November 2017 Available from: https://ctep-cancer-gov.rpci.idm.oclc.org/protocoldevelopment/electronic_applications/docs/CTCAE_v5_Quick_Reference_8.5x11.pdf (Accessed on March 09, 2018). 14_QuickReference_85x11.pdf (last access: April 14, 2022)

*One patient had hyperbilirubinemia attributed to alpelisib. Bold values refer to the number of patients with grade 3 adverse events

Overall, 23/27 (85.19%) patients discontinued alpelisib of which 11/23 (47.83%) discontinued alpelisib due to AEs (**Table 4**). 14 (51.9%) patients required dose interruption and reduction. The median time on alpelisib in patients who developed AEs was 2.75 months (range: < 1.00, 10.43 months), while the median time on alpelisib in those who did not experience adverse events was 5.54 months (range: 1.15, 5.77). Median duration of therapy was 2 months (range: < 1.00, 8.30) in patients who experienced grade 3 AEs and 6.28 months (range: 1.15, 10.43) in those who did not develop grade 3 AEs. There was no association between clinical/demographic/pathological/molecular characteristics with AEs (**Supplementary Table 1**).

**Table 4:** Alpelisib treatment details.

Variables		N (%)
Sequence of alpelisib	2^nd^ line	6 (22.2%)
	3^rd^ line	7 (25.9%)
	4^th^ line	4 (14.8%)
	5^th^ line and beyond	10 (37.0%)
Median dose at initiation (mg) (range)		300 (150-300)
RDI median (%) (range)		0.94 (0.32-1.00)
Median duration on alpelisib (months) (range)	Total	2.75 (0-10.43)
	AEs group	2.57 (0-10.43)
	No AEs group	5.54 (1.15-5.77)
**Dose Interruptions and Discontinuations N (%)**		
No dose reductions		13 (48.2%)
Dose reductions/interruptions due to AEs		14 (51.9%)
Discontinuation		23 (85.18%)
Reason for discontinuation	AEs	11 (47.8%)
	Progression of disease	12 (52.2%)

RDI, relative dose intensity.

### Efficacy analysis

**Figure 1:**
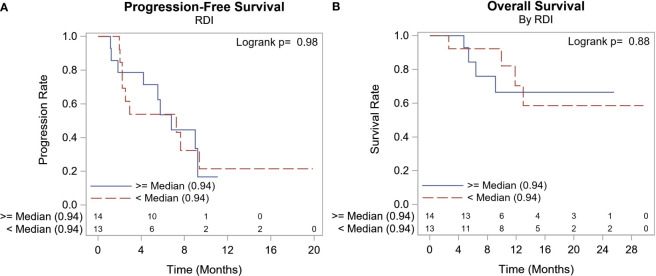
Outcome Survival Summary. **(A)** Median PFS was 6.8 months with 95% CI (2.5, 9.2). **(A)** 8/27 (29.6%) patients had died, and all deaths were attributed to metastatic breast cancer. The median survival had not been reached, and the 12-month OS/disease-specific survival (DSS) rate was 67.7%.

3/27 (11.11%) patients discontinued therapy before response assessment due to grade 3 AEs. ORR was 12.5% (3/24) and these were all PR, no CR was observed. Median PFS was 6.8 months with 95% CI (2.5, 9.2) (**Figure 1A**). The median duration of response was 5.77 months (range: 5.54, 8.98). At the time of this report, 8/27 (29.6%) patients had died, and all deaths were attributed to metastatic breast cancer. The median survival had not been reached, 12-month OS/disease-specific survival (DSS) rate was 67.7% (range: 43.0%, 83.5%) (**Figure 1B**).

**Figure 2:**
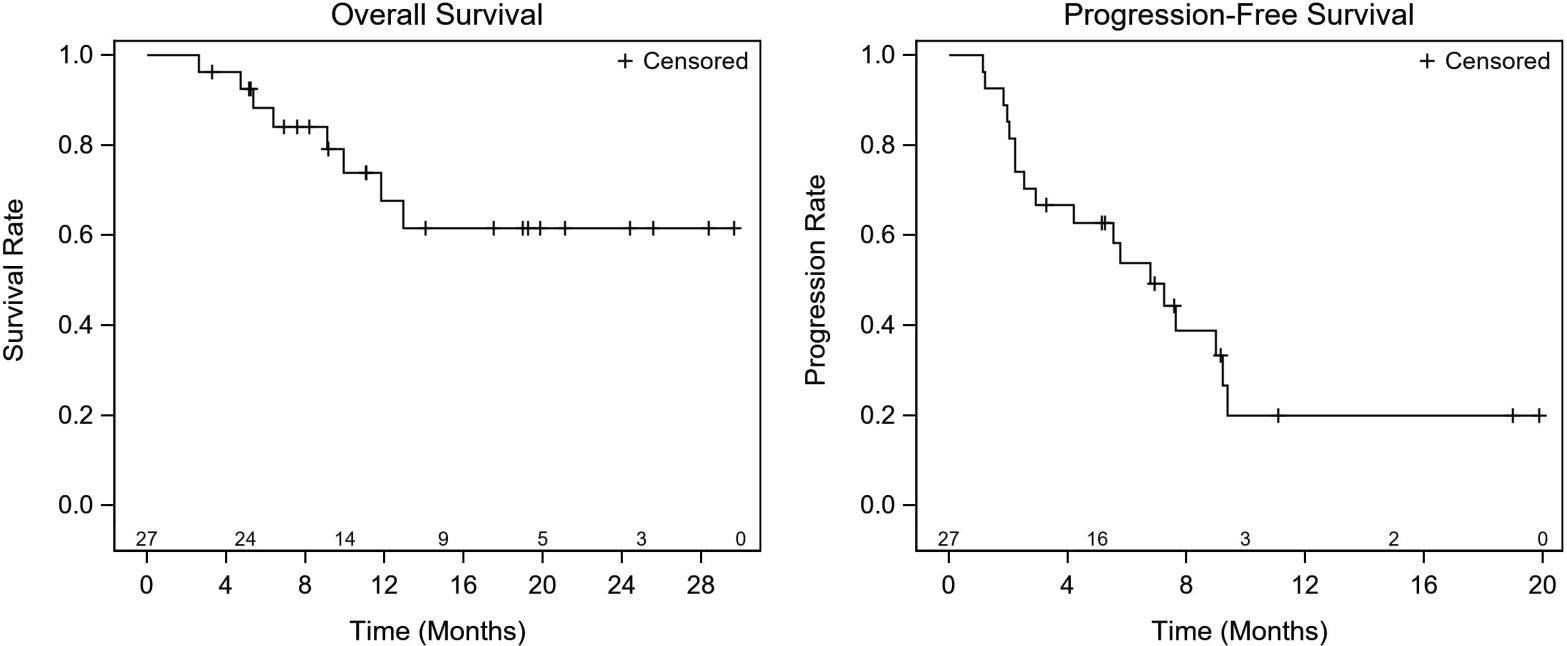
RDI & Survival Summary. There was no statistically significant difference in RDI in terms of the OS or PFS.

To investigate if there are any clinical, laboratory, or molecular predictors of treatment response to alpelisib, we investigated the association between these factors and responses. **Table 5** summarizes study characteristics by best response (PR, SD, and PD). Generally, those with PD as the best response had higher WBC of 8.05 10^9^/L (range: 4.80, 16.20 10^9^/L), p=0.029, and ALP at baseline 133.0 IU/L (range: 77.00, 725.0 IU/L), p=0.046, more nonmetabolic comorbidities, a higher number of liver metastasis, PIK3CA E545K mutations, and a shorter duration on alpelisib compared to those with PR and SD. The median RDI was 100% in patients who had documented response (range: 94%, 100%) and 77% (range: 32%, 100%) in patients who did not have documented response. There was no statistically significant difference in RDI in terms of the OS or PFS (**Figure 2**).

**Table 5:** Association of clinical/demographic characteristics with best response to alpelisib.

				PR	SD	PD	P-value
			N	3 (12.5)	14 (58.3)	7 (29.2)	
Gender			Female	3 (12.5%)	14 (58.3%)	7 (29.2%)	
Race			White	3 (14.3%)	12 (57.1%)	6 (28.6%)	0.820
			Black			1 (100.0%)	
			Hispanic		1 (100.0%)		
Age at initiation of alpelisib (years)			Median (range)	68.91 (66.17-73.31)	66.00 (50.17-77.32)	67.13 (51.26-76.84)	0.747
BMI			Median (range)	23.80 (22.70-42.10)	26.70 (19.40-34.00)	25.60 (19.80-38.10)	0.966
ECOG PS score			0	2 (14.3%)	9 (64.3%)	3 (21.4%)	0.729
			1	1 (10.0%)	5 (50.0%)	4 (40.0%)	
Comorbidities			No	2 (28.6%)	3 (42.9%)	2 (28.6%)	0.363
			Yes	1 (5.9%)	11 (64.7%)	5 (29.4%)	
	Type 2 diabetes		No	3 (13.6%)	13 (59.1%)	6 (27.3%)	1.000
			Yes		1 (50.0%)	1 (50.0%)	
	Hypertension		No	3 (20.0%)	7 (46.7%)	5 (33.3%)	0.357
			Yes		7 (77.8%)	2 (22.2%)	
	Dyslipidemia		No	2 (10.5%)	12 (63.2%)	5 (26.3%)	0.475
			Yes	1 (20.0%)	2 (40.0%)	2 (40.0%)	
	Obesity		No	3 (12.5%)	14 (58.3%)	7 (29.2%)	
	Cardiovascular disease		No	3 (13.0%)	13 (56.5%)	7 (30.4%)	1.000
			Yes		1 (100.0%)		
	Thyroid disease		No	3 (15.0%)	11 (55.0%)	6 (30.0%)	1.000
			Yes		3 (75.0%)	1 (25.0%)	
	Chronic lung disease		No	3 (13.6%)	13 (59.1%)	6 (27.3%)	1.000
			Yes		1 (50.0%)	1 (50.0%)	
	*Nonmetabolic comorbidities		No	3 (15.8%)	13 (68.4%)	3 (15.8%)	0.022
			Yes		1 (20.0%)	4 (80.0%)	
Number of organs with metastasis			1		2 (100.0%)		0.814
			2	2 (16.7%)	5 (41.7%)	5 (41.7%)	
			3	1 (12.5%)	5 (62.5%)	2 (25.0%)	
			4		2 (100.0%)		
Bone metastasis			No		1 (100.0%)		1.000
			Yes	3 (13.0%)	13 (56.5%)	7 (30.4%)	
Brain metastasis			No	2 (9.5%)	13 (61.9%)	6 (28.6%)	0.505
			Yes	1 (33.3%)	1 (33.3%)	1 (33.3%)	
Liver metastasis			No	1 (7.7%)	11 (84.6%)	1 (7.7%)	0.015
			Yes	2 (18.2%)	3 (27.3%)	6 (54.5%)	
Lung metastasis			No	3 (15.8%)	9 (47.4%)	7 (36.8%)	0.195
			Yes		5 (100.0%)		
Visceral metastasis			No	1 (12.5%)	3 (37.5%)	4 (50.0%)	0.276
			Yes	2 (12.5%)	11 (68.8%)	3 (18.8%)	
Histology			IDC	1 (20.0%)	3 (60.0%)	1 (20.0%)	0.405
			ILC	1 (12.5%)	5 (62.5%)	2 (25.0%)	
			Poorly differentiated		6 (60.0%)	4 (40.0%)	
			Mixed	1 (100.0%)			
Type of molecular test			Tissue molecular test	1 (8.3%)	6 (50.0%)	5 (41.6%)	0.321
			Guardant 360 (peripheral blood)	2 (16.7%)	8 (66.7%)	2 (16.7%)	
PIK3CA H1047R mutation			No	1 (8.3%)	5 (41.7%)	6 (50.0%)	0.084
			Yes	2 (16.7%)	9 (75.0%)	1 (8.3%)	
PIK3CA E545K mutation			No	2 (12.5%)	12 (75.0%)	2 (12.5%)	0.022
			Yes	1 (12.5%)	2 (25.0%)	5 (62.5%)	
PIK3CA E546K mutation			No	3 (13.0%)	14 (60.9%)	6 (26.1%)	0.417
			Yes			1 (100.0%)	
PIK3CA E542K mutation			No	2 (9.5%)	12 (57.1%)	7 (33.3%)	0.215
			Yes	1 (33.3%)	2 (66.7%)		
PIK3CA E418K mutation			No	3 (13.0%)	13 (56.5%)	7 (30.4%)	1.000
			Yes		1 (100.0%)		
PIK3CA C420R mutation			No	3 (13.0%)	13 (56.5%)	7 (30.4%)	1.000
			Yes		1 (100.0%)		
PIK3CA D350N mutation			No	3 (13.0%)	13 (56.5%)	7 (30.4%)	1.000
			Yes		1 (100.0%)		
Alpelisib sequence in relation to treatments received			2nd Line	1 (20.0%)	3 (60.0%)	1 (20.0%)	0.558
			3rd Line	1 (20.0%)	4 (80.0%)		
			4th Line		3 (75.0%)	1 (25.0%)	
			5th Line and Beyond	1 (10.0%)	4 (40.0%)	5 (50.0%)	
Dose initiation (mg)			Median(range)	300 (300-300)	300 (150-300)	300 (300-300)	0.700
Dose reduction/Interruption			No	2 (20.0%)	6 (60.0%)	2 (20.0%)	0.515
			Yes	1 (7.1%)	8 (57.1%)	5 (35.7%)	
Discontinuation			No	3 (18.8%)	9 (56.3%)	4 (25.0%)	0.686
			Yes		5 (62.5%)	3 (37.5%)	
Number of prior systemic therapies			Median	2(1-7)	3(1-6)	5 (1-6)	0.417
Time on alpelisib (months)			Median (range)	5.77 (5.54-8.98)	7.05 (1.97-10.43)	1.80 (1.15-2.75)	0.004
Prior treatment		CDK4/6 +AI	No	1 (14.3%)	3 (42.9%)	3 (42.9%)	0.575
			Yes	2 (11.8%)	11 (64.7%)	4 (23.5%)	
		CDK4/6/ Fulvestrant	No	2 (12.5%)	9 (56.3%)	5 (31.3%)	1.000
		Yes	1 (12.5%)	5 (62.5%)	2 (25.0%)	
		Xeloda	No	2 (16.7%)	9 (75.0%)	1 (8.3%)	0.084
		Yes	1 (8.3%)	5 (41.7%)	6 (50.0%)	
		Anthracycline	No	2 (9.1%)	14 (63.6%)	6 (27.3%)	0.163
		Yes	1 (50.0%)		1 (50.0%)	
		Taxane	No	2 (12.5%)	11 (68.8%)	3 (18.8%)	0.276
		Yes	1 (12.5%)	3 (37.5%)	4 (50.0%)	
		Platinum	No	3 (12.5%)	14 (58.3%)	7 (29.2%)	.
		Yes				
		Everolimus/AI	No	1 (7.7%)	8 (61.5%)	4 (30.8%)	0.856
		Yes	2 (18.2%)	6 (54.5%)	3 (27.3%)	
		A/C	No	3 (13.0%)	13 (56.5%)	7 (30.4%)	1.000
		Yes		1 (100.0%)		
		Eribulin	No	2 (9.1%)	13 (59.1%)	7 (31.8%)	0.315
		Yes	1 (50.0%)	1 (50.0%)		
		Gemcitabine	No	3 (12.5%)	14 (58.3%)	7 (29.2%)	.
		Yes				
		Single agent AI/SERD	No	2 (13.3%)	9 (60.0%)	4 (26.7%)	1.000
		Yes	1 (11.1%)	5 (55.6%)	3 (33.3%)	
		Olaparib	No	3 (13.6%)	12 (54.5%)	7 (31.8%)	0.645
		Yes		2 (100.0%)		
		Clinical Trial	No	3 (13.6%)	14 (63.6%)	5 (22.7%)	0.163
		Yes			2 (100.0%)	
Number of prior lines			Median (range)	2.00 (1-7)	3.00 (1-6)	4.00 (1-5)	0.449
RDI			Median (range)	1.00 (0.94-1.00)	0.78 (0.45-1.00)	0.65 (0.32-1.00)	0.232
Baseline WBC (10^9^/L)			Median (range)	4.53 (3.16-8.96)	4.53 (2.10-6.68)	8.05 (4.80-16.20)	0.029
Baseline ALP(IU/L)			Median (range)	103.00 (90.00-119.00)	87.00 (21.00-155.00)	133.00 (77.00-725.00)	0.046

PR, partial response; PD, progressive disease; SD, stable disease; BMI, Body mass index; ECOG PS scale, Eastern Cooperative Oncology Group performance status scale; IDC, invasive ductal carcinoma; ILC, invasive lobular carcinoma; CDK4/6 inhibitors, cyclin-dependent kinase 4/6 inhibitors; AI, aromatase inhibitors; A/C, anthracycline and cyclophosphamide; SERD, selective estrogen receptor degrader; RDI, relative dose intensity; WBC, white blood cells; ALP, alkaline phosphatase.

*Nonmetabolic comorbidities: one patient had history of venous thromboembolism; one patient had transient ischemic attack; one patient had leukemia; one patient with gastroesophageal disease.

### Mutation analysis

14/27 (52%) patients had PIK3CA detected in peripheral blood (Guardant 360 testing), and 13/27 (48%) had PIK3CA detected on tumor tissue (Omniseq, Foundation One). PIK3CA H1047R was the most common mutation subtype identified in 14/27 (51.9%) patients, followed by PIK3CA E545K in 9/27 (33.3%) patients and PIK3CA E542K in 3 (11.1%) patients (**Table 1**). Of note, 3 patients had double PIK3CA mutations.

## Discussion

Our study expands upon the rapidly accumulating reports of real-world experience with alpelisib outside a clinical trial (12–18). We report a low ORR to alpelisib at 12.5% in a cohort of heavily pretreated patients with a median of 3 lines of prior treatment including chemotherapy, with rather a brief duration of response with a median of 5.77 months.

Alpelisib was administered in the second line in SOLAR1 and reported a 26% ORR, which is two times higher than the finding in our study. The patient selection and the fact that our cohort comprises mostly heavily pretreated patients may account for the variation observed in efficacy between SOLAR1 results and ours. BYLieve study which enrolled patients who are probably more comparable to our patients, reported a response rate of 17% with a median duration of response of 6.6 months, all of which were PR which is consistent with our study findings (3–6). Furthermore, we show a median PFS of 6.8 months, which is comparable to the 5.7 months reported by the BYLieve study (5, 6). Alpelisib is approved for treatment in the second-line metastatic setting. However, in our study, a large proportion of patients were heavily pretreated, likely due to an attempt to derive benefit from this medication once FDA approval was obtained. As previously shown, the benefit is modest at best and consistent with another case series that reported a PFS benefit of 5.5 months for patients who had a median of ≥ 3 prior treatments (18).

We report a significant rate of alpelisib-induced AEs with 89% of patients reporting at least one AE of any grade considered to be treatment-related. The frequency of AEs in our study is higher than what was observed in the published clinical trials of alpelisib; SOLAR1 and BYLieve. In our study, the most common AEs observed were hyperglycemia 16/27 (59.3%), nausea 11 (40.7%), diarrhea 10 (37.0%) fatigue 7 (25.9%), rash 6 (22.2%). The clinical trials reported the overall rate of any grade hyperglycemia in the range of 60-64%, diarrhea 58%-60%, nausea 45%, decreased appetite 30-35%, and rash 30-35% (3–6, 19). Once again, this variation could be explained by our study population being heavily pretreated. In a similar study by Miller et al. describing the real-world experience of alpelisib, they also observed higher AEs than what was reported in the clinical trials (Hyperglycemia- 66.7%, rash- 45.5%, diarrhea- 72.7%) (18). Moreover, hyperglycemia, rash, and GI AEs were the most common grade 3 AEs. Grade 3 AEs occurred considerably more frequently in our study population than in previously reported clinical trials, hyperglycemia (53% vs 25-33%), rash (15% vs 10%), diarrhea (15% vs 4.0 -7.0%) (3–5). Clearly, data shows that hyperglycemia is a significant AE experienced by a large majority of patients on alpelisib. The multiple case reports of life-threatening diabetic ketoacidosis with alpelisib are cause for considerable concern (20–23). In the elderly population, this poses a serious challenge because patients have to self-monitor and take additional medications, in addition to dealing with symptoms including polyuria, dehydration, and dizziness that could increase the risk of falls and injuries (16). In a study by Almodallal and colleagues that examined the tolerance of alpelisib in a heavily pretreated elderly population (median age 71 years), 15/35 (43%) patients discontinued alpelisib due to AEs (14). This also supports the fact that alpelisib would be harder to tolerate for elderly patients.

Dose interruptions/reductions were required in 14/27 (51.9%) patients. Another real-world study reported similarly high rates of dose reductions due to AEs (12). These findings highlight the challenge associated with alpelisib as it is believed that the best therapeutic benefit of alpelisib is attained by maintaining a high median dosage intensity, as was shown in exposure-efficacy studies (19). Our findings, along with those of other real-world data, contradict the acceptable tolerability reported in clinical trials of alpelisib, where the rate of discontinuation due to side effects ranged from 21% to 25% (3–6).

The FDA label does not limit the use of alpelisib based on past therapy. It is important to highlight that 40% of patients in our study had experienced disease progression on a prior mTOR inhibitor (everolimus) before alpelisib. This may have influenced the response and the observed increased toxicity from alpelisib since it is well established that mTOR is a key downstream player in the PI3K-AKT pathway (24). Furthermore, 30% of patients had already received fulvestrant before treatment with alpelisib in combination with fulvestrant, a population of patients that would have been excluded from the SOLAR-1 trial. Therefore, caution must be exercised when data from clinical trials (where stringent inclusion and exclusion criteria are used) are extrapolated to the general population. This knowledge gap can be readily filled by real world studies like the one we report here.

Given the increased toxicity of alpelisib, cautious selection of patients is important. In our small cohort, patients with liver metastases responded poorly to alpelisib. That is in contrast to the SOLAR1 subgroup analysis, which showed that individuals with liver metastasis had a survival benefit with alpelisib. SOLAR1 also suggested that alpelisib could perform effectively for those with a higher disease burden. In our study, patients with higher level of ALP at baseline which we believe is associated with increased disease burden, continued to experience disease progression on alpelisib. Our study, given the small sample size, is not powered to test these variables as predictive factors of response to alpelisib, but clearly, in consistency with prior literature, these observations are of possible prognostic value. Patient-reported outcome measure (PROM) data from the SOLAR-1 clinical trial, reported that patients who received alpelisib did not experience a significant decline in overall health-related quality of life (25). In our real-world report, patients did experience higher rates of AEs such as diarrhea, appetite loss, nausea or vomiting, and fatigue that could potentially impact their social functioning. This difference suggests that in the real world, alpelisib may have a negative effect on the patient’s quality of life as opposed to the observation on clinical trial. This highlights the importance of conducting and reporting real-word studies once new treatments are approved. This helps to accurately gauge the efficacy and safety of new treatments at a population level with less stringent clinical characteristics compared to patients on the clinical trials, that lead to the drug approvals. This knowledge would aid in preventing unintended consequences of treatment and in choosing therapies that are consistent with the agreed-upon goals of metastatic cancer therapy being palliative and improving quality of life (26). It is difficult to ensure patient adherence when they are experiencing harmful adverse events and this report highlights the practical challenges and limitations of alpelisib utilization in real-world. Given the rapidly evolving treatment landscape of HR-positive metastatic breast cancer, future research must focus on the optimal sequencing of agents to maintain good clinical outcomes as well as good quality of life, particularly given the recent data on the efficacy and tolerance of newer antibody-drug conjugates (trastuzumab deruxtecan and sacituzumab govitecan) (27, 28).

Finally, we acknowledge the limitations of our descriptive study, which include the small sample size, and retrospective design that introduces selection bias and does not accurately measure efficacy endpoints such as PFS or ORR in addition to being a single arm study lacking a comparison arm. Additionally, unlike clinical trials, response assessment in our study was not done at consistent intervals, as is common in real-world practice. Nonetheless, our results are consistent with other similar real-world reports. In conclusion, it is critical to utilize and incorporate rapidly accumulating real-world data on the effectiveness and toxicity of alpelisib in addition to data from clinical trials when making treatment decisions for our patients.

## Data availability statement

The original contributions presented in the study are included in the article/**Supplementary Material**. Further inquiries can be directed to the corresponding author.

## Ethics statement

The studies involving human participants were reviewed and approved by The Roswell Park Institutional Review Board (IRB). Written informed consent for participation was not required for this study in accordance with the national legislation and the institutional requirements.

## Author contributions

Conceptualization, SA and SG; validation, SG and AR; data collection, SA and AR; statistical analysis, AG and KA; writing-original draft preparation, SA and AR; writing-editing, SG, AE, TO’C, and EL; supervision, SG. All authors have read and agreed to the published version of the manuscript.

## Funding

Research reported in this publication was supported by the National Center for Advancing Translational Sciences of the National Institutes of Health under award Number KL2TR001413 and UL1TR001412. The content is solely the responsibility of the authors and does not necessarily represent the official views of the NIH.

## Conflict of interest

The authors declare that the research was conducted in the absence of any commercial or financial relationships that could be construed as a potential conflict of interest.

## Publisher’s note

All claims expressed in this article are solely those of the authors and do not necessarily represent those of their affiliated organizations, or those of the publisher, the editors and the reviewers. Any product that may be evaluated in this article, or claim that may be made by its manufacturer, is not guaranteed or endorsed by the publisher.
