# Supplementary material for: Real world outcomes with alpelisib in metastatic hormone receptor-positive breast cancer patients: A single institution experience
Source: Front Oncol. 2022 Oct 20;12:1012391. doi: 10.3389/fonc.2022.1012391 (PMC9631302; doi:10.3389/fonc.2022.1012391)
Supplement: Supplementary file 1 [file Table_1.docx]

Supplementary Material

**Supplementary tables:**

**Table 1: Association of** **Clinical/Demographic Characteristics with Adverse Events (AEs)**

|  | | | **No AE** | **AE** | **p-value** |
| --- | --- | --- | --- | --- | --- |
|  | | N | 3 (11.1) | 24 (88.9) |  |
| Gender | | Female | 3 (11.1%) | 24 (88.9%) |  |
| Race | | White | 2 (8.7%) | 21 (91.3%) | 0.395 |
|  |  | Black | 1 (100.0%) |  |  |
|  |  | Hispanic |  | 1 (100.0%) |  |
|  |  | Asian |  | 1 (100.0%) |  |
|  |  | Other |  | 1 (100.0%) |  |
| Age at initiation of alpelisib | | Mean/Std/N | 69.20/3.19/3 | 63.49/10.30/24 | 0.418 |
|  |  | Median (range) | 68.91 (66.17-72.54) | 66.00 (43.70-77.32) |  |
| BMI | | Mean | 28.77 | 26.57 | 0.748 |
|  |  | Median (range) | 67.42 (64.58-72.37) | 59.50 (42.79-76.09) |  |
| ECOG performance score | | 0 | 2 (13.3%) | 13 (86.7%) | 1.000 |
|  |  | 1 | 1 (8.3%) | 11 (91.7%) |  |
| Comorbidities | | No | 2 (22.2%) | 7 (77.8%) | 0.250 |
|  |  | Yes | 1 (5.6%) | 17 (94.4%) |  |
|  | Type 2 Diabetes | No | 3 (12.0%) | 22 (88.0%) | 1.000 |
|  |  | Yes |  | 2 (100.0%) |  |
|  | Hypertension | No | 3 (16.7%) | 15 (83.3%) | 0.529 |
|  |  | Yes |  | 9 (100.0%) |  |
|  | Dyslipidemia | No | 3 (13.6%) | 19 (86.4%) | 1.000 |
|  |  | Yes |  | 5 (100.0%) |  |
|  | Obesity | No | 3 (11.1%) | 24 (88.9%) |  |
|  | Cardiovascular disease | No | 3 (12.0%) | 22 (88.0%) | 1.000 |
|  |  | Yes |  | 2 (100.0%) |  |
|  | Thyroid disease | No | 3 (13.0%) | 20 (87.0%) | 1.000 |
|  |  | Yes |  | 4 (100.0%) |  |
|  | Chronic lung disease | No | 3 (12.0%) | 22 (88.0%) | 1.000 |
|  |  | Yes |  | 2 (100.0%) |  |
|  | Nonmetabolic comorbidities | No | 2 (9.1%) | 20 (90.9%) | 0.474 |
|  |  | Yes | 1 (20.0%) | 4 (80.0%) |  |
| Number of organs with metastasis | | 1 |  | 2 (100.0%) | 0.480 |
|  |  | 2 | 3 (23.1%) | 10 (76.9%) |  |
|  |  | 3 |  | 9 (100.0%) |  |
|  |  | 4 |  | 3 (100.0%) |  |
| Bone metastasis | | No |  | 1 (100.0%) | 1.000 |
|  |  | Yes | 3 (11.5%) | 23 (88.5%) |  |
| Brain metastasis | | No | 3 (12.5%) | 21 (87.5%) | 1.000 |
|  |  | Yes |  | 3 (100.0%) |  |
| Liver metastasis | | No | 1 (7.1%) | 13 (92.9%) | 0.596 |
|  |  | Yes | 2 (15.4%) | 11 (84.6%) |  |
| Lung metastasis | | No | 3 (14.3%) | 18 (85.7%) | 1.000 |
|  |  | Yes |  | 6 (100.0%) |  |
| Visceral metastasis | | No | 2 (25.0%) | 6 (75.0%) | 0.201 |
|  |  | Yes | 1 (5.3%) | 18 (94.7%) |  |
| Prior best response | | PR | 1 (16.7%) | 5 (83.3%) | 0.545 |
|  |  | SD | 2 (9.5%) | 19 (90.5%) |  |
| Longest prior duration of response | | Mean/Std/N | 10.67/2.31/3 | 35.55/28.48/22 | 0.064 |
|  |  | Median (range) | 12.00 (8.00-12.00) | 24.00 (4.00-114.00) |  |
| Histology | | IDC |  | 6 (100.0%) | 0.137 |
|  |  | ILC | 1 (12.5%) | 7 (87.5%) |  |
|  |  | Poorly differentiated | 1 (8.3%) | 11 (91.7%) |  |
|  |  | Mixed | 1 (100.0%) |  |  |
| Type of molecular test | | Omniseq (Tissue) |  | 7 (100.0%) | 0.395 |
|  |  | Foundation one (Tissue) |  | 6 (100.0%) |  |
|  |  | Guardant 360 (Blood) | 3 (21.4%) | 11 (78.6%) |  |
| PIK3CA H1047R mutation | | No |  | 13 (100.0%) | 0.222 |
|  |  | Yes | 3 (21.4%) | 11 (78.6%) |  |
| PIK3CA E545K mutation | | No | 2 (11.1%) | 16 (88.9%) | 1.000 |
|  |  | Yes | 1 (11.1%) | 8 (88.9%) |  |
| PIK3CA E546K mutation | | No | 3 (11.5%) | 23 (88.5%) | 1.000 |
|  |  | Yes |  | 1 (100.0%) |  |
| PIK3CA E542K mutation | | No | 3 (12.5%) | 21 (87.5%) | 1.000 |
|  |  | Yes |  | 3 (100.0%) |  |
| PIK3CA E418K mutation | | No | 3 (11.5%) | 23 (88.5%) | 1.000 |
|  |  | Yes |  | 1 (100.0%) |  |
| PIK3CA C420R mutation | | No | 3 (11.5%) | 23 (88.5%) | 1.000 |
|  |  | Yes |  | 1 (100.0%) |  |
| PIK3CA D350N mutation | | No | 3 (11.5%) | 23 (88.5%) | 1.000 |
|  |  | Yes |  | 1 (100.0%) |  |
| Line of therapy | | 2nd Line | 1 (16.7%) | 5 (83.3%) | 0.345 |
|  |  | 3rd Line | 1 (14.3%) | 6 (85.7%) |  |
|  |  | 4th Line | 1 (25.0%) | 3 (75.0%) |  |
|  |  | 5th Line and Beyond |  | 10 (100.0%) |  |
| Dose initiation (mg) | | Mean | 300 | 293 | 0.814 |
|  |  | Median (range) | 300 (300-300) | 300 (150-300) |  |
| Dose reduction | | No | 3 (25.0%) | 9 (75.0%) | 0.085 |
|  |  | Yes |  | 14 (100.0%) |  |
| Interruption | | No | 3 (23.1%) | 10 (76.9%) | 0.098 |
|  |  | Yes |  | 14 (100.0%) |  |
| Discontinuation | | No | 3 (18.8%) | 13 (81.3%) | 0.248 |
|  |  | Yes |  | 11 (100.0%) |  |
| Number of prior systemic therapies | | Median (range) | 2 (1-3) | 3 (1-7) | 0.256 |
| Time on alpelisib (months) | | Mean | 4.15 | 4.31 | 0.819 |
|  |  | Median (range) | 5.54 (1.15-5.77) | 2.57 (0.00-10.43) |  |
| Prior treatment: | CDK4/6 +AI | No |  | 7 (100.0%) | 0.545 |
|  |  | Yes | 3 (15.0%) | 17 (85.0%) |  |
|  | CDK4/6/Fulvestrant | No | 3 (15.8%) | 16 (84.2%) | 0.532 |
|  |  | Yes |  | 8 (100.0%) |  |
|  | Xeloda | No | 2 (13.3%) | 13 (86.7%) | 1.000 |
|  |  | Yes | 1 (8.3%) | 11 (91.7%) |  |
|  | Anthracycline | No | 3 (12.0%) | 22 (88.0%) | 1.000 |
|  |  | Yes |  | 2 (100.0%) |  |
|  | Taxane | No | 2 (11.8%) | 15 (88.2%) | 1.000 |
|  |  | Yes | 1 (10.0%) | 9 (90.0%) |  |
|  | Platinum | No | 3 (11.5%) | 23 (88.5%) | 1.000 |
|  |  | Yes |  | 1 (100.0%) |  |
|  | Everolimus/AI | No | 2 (12.5%) | 14 (87.5%) | 1.000 |
|  |  | Yes | 1 (9.1%) | 10 (90.9%) |  |
|  | A/C | No | 3 (11.5%) | 23 (88.5%) | 1.000 |
|  |  | Yes |  | 1 (100.0%) |  |
|  | Eribulin | No | 3 (12.0%) | 22 (88.0%) | 1.000 |
|  |  | Yes |  | 2 (100.0%) |  |
|  | Gemcitabine | No | 3 (11.5%) | 23 (88.5%) | 1.000 |
|  |  | Yes |  | 1 (100.0%) |  |
|  | Single agent AI/SERD | No | 3 (16.7%) | 15 (83.3%) | 0.529 |
|  |  | Yes |  | 9 (100.0%) |  |
|  | Olaparib | No | 3 (12.0%) | 22 (88.0%) | 1.000 |
|  |  | Yes |  | 2 (100.0%) |  |
|  | Clinical Trial | No | 3 (12.0%) | 22 (88.0%) | 1.000 |
|  |  | Yes |  | 2 (100.0%) |  |
| Number of prior lines | | Median (range) | 2 (1-3) | 3 (1-7) | 0.250 |
| RDI | | Median (range) | 1.00 (1.00-1.00) | 0.78 (0.32-1.00) | 0.093 |
| Best response | | PR | 2 (66.7%) | 1 (33.3%) | 0.021 |
|  |  | SD |  | 14 (100.0%) |  |
|  |  | PD | 1 (14.3%) | 6 (85.7%) |  |
|  |  | Not assessed |  | 3 (100.0%) |  |
| Objective response rate | | Yes (CR+PR) | 2 (66.7%) | 1 (33.3%) | 0.032 |
|  |  | No (Everything Else) | 1 (4.8%) | 20 (95.2%) |  |

Abbreviations: PR: partial response; PD: progressive disease; SD: stable disease; BMI: Body mass index; ECOG PS scale: Eastern Cooperative Oncology Group performance status scale; IDC: invasive ductal carcinoma; ILC: invasive lobular carcinoma; CDK4/6 inhibitors: cyclin-dependent kinase 4/6 inhibitors; AI: aromatase inhibitors; A/C: anthracycline and cyclophosphamide; SERD: selective estrogen receptor degrader; RDI: relative dose intensity; WBC: white blood cells; ALP: alkaline phosphatase;

*Nonmetabolic comorbidities: one patient had history of venous thromboembolism; one patient had transient ischemic attack; one patient had leukemia; one patient with gastroesophageal disease.
